# Prevalence of Zoonotic Intestinal Helminths of Canids in Moghan Plain, Northwestern Iran

**Published:** 2010-06

**Authors:** M Zare-Bidaki, I Mobedi, S Sadeghieh Ahari, S Habibizadeh, SR Naddaf, MR Siavashi

**Affiliations:** 1 Department of Microbiology, School of Medicine, Rafsanjan University of Medical Sciences, Iran; 2 Department of Medical Parasitology and Mycology, School of Public Health, Tehran University of Medical Science, Tehran, Iran; 3 Department of Pediatrics and Social Medicine, School of Medicine, Ardebil University of Medical Sciences, Iran; 4 Department of Infectious Disease, School of Medicine, Ardebil University of Medical Sciences, Iran; 5 Department of Parasitology, Pasteur Institute of Iran, Tehran, Iran

**Keywords:** Zoonotic helminths, Canids, Iran

## Abstract

**Background:**

The present study was aimed to elucidate the status of intestinal helminth infections in canids of Moghan Plain, northwestern Iran.

**Methods:**

Eighty-five intestine samples from dead or shot wild canids, 59 fecal samples from sheepdogs and 5 from red foxes were collected from 2006 to 2008 and examined in Parasitology department of Pasteur Institute of Iran.

**Results:**

Generally, adult worms, larvae, and eggs of 13 species of various parasitic helminths were recovered. Necropsy examinations showed that 96.47% animals harbored at least one helminth species. The prevalence of different species in necropsy were *Mesocestoides* sp. 84.7%, *Rictolaria* spp. 55.3%, *Macranthorhynchus hirudinaceus* 45.9%, *Toxocara canis* 43.5%, *Toxascaris* spp. 35.3%, *Joyeuxiella* sp. 34.1%; hookworms; 22.4%, *Taenia* spp. 11.8%, *Alaria* spp. 2.4% and *Dipylidium caninum* 1.2%. Besides, eggs belonging to 10 species of parasitic helminths were identified in 46 fecal samples and generally, 30.9% of samples harbored eggs of at least one helminth species.

**Conclusion:**

The high prevalence of various helminth infections among canids in Moghan plain and contamination of environment by helminths eggs may increase the risk of infection for native people.

## Introduction

Wild and domestic animals are considered as main sources of emerging human and domestic livestock pathogens and zoonoses of public health significance (1). In Iran, four categories of canids, namely feral or stray dogs, working sheepdogs, pet dogs and wild canids (especially red foxes and jackals) are known as reservoirs of numerous zoonotic diseases. There are some reports on prevalence of intestinal helminths among canids from different parts of Iran (2–8). Due to relatively high annual precipitation, building of dams on Aras River, huge irrigations networks and agriculture plans Moghan plain has become one of the most important areas for farming and stockbreeding in Iran, where nomadic, rural, and urban inhabitants come into close contacts with domestic and wild canids. There were two reports on the prevalence of helminthic infections in the carnivores of this area dating back to 1973 and 1993 (3, 5). Since then, intensive economic and social alterations including rapid population growth and development of villages and cities have resulted to major ecological changes in Moghan Plain, Iran (9).

The present study was conducted to elucidate the status of intestinal helminth infections among canids in this area.

## Materials and Methods

### Study area

**Fig. 1:**
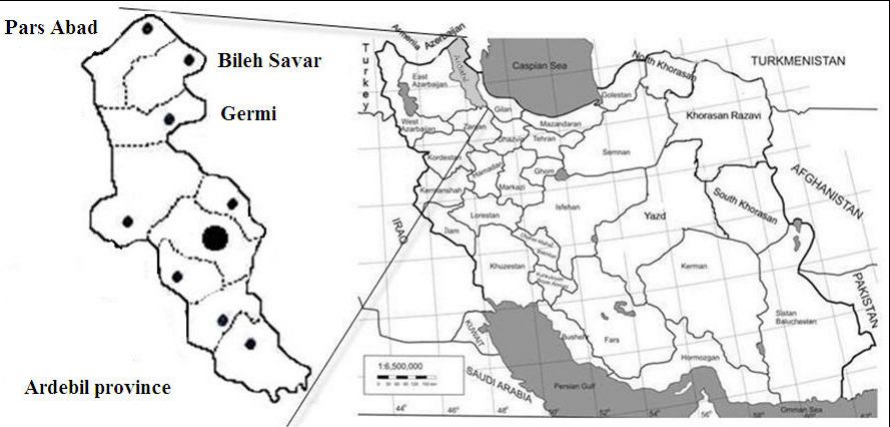
Map of Moghan Plain, the study area in northern part of Ardebil Province comprises three counties; Pars Abad, Bileh Savar and Germi

This study was performed in the Moghan Plain (local name: Dasht-e-Moghan), situated in the northernmost parts of the Ardebil Province, northwestern Iran (Fig. 1). The area comprises three counties including Pars Abad, Bileh Savar and Germi covering an area of nearly 5245 km^2^ with a total population of approximately 310,000. The area covers the low landing areas with altitude of 32m up to the plains of 1023 m high with mean annual precipitation of 223 mm. The area is bordered with Azerbaijan Republic to the north and east with longitudes and latitudes ranges of approximately 46°52'53?E–48°21'30? E and 39° 0' 0?N –39°36'20?N, respectively. The inhabitants are mainly of Azeri ethnic group and mostly practice farming and stockbreeding.

### Samples

From September 2006 to November 2008, 149 samples were collected from different localities in the study area. Eighty five intestine samples were obtained from dead or shot wild canids comprising 84 red foxes (*Vuleps vulpes*) and one golden jackal (*Canis aureus*). The animal carcasses were autopsied; small intestines were tied off at both ends and removed. The fecal samples were also collected from the animals' rectum as well. Besides, five environmentally deposited feces from vicinity of red foxes dens and 59 fecal samples from working sheepdogs (*Canis lupus*.*familiaris*) were collected and stored in 70% ethanol. All Samples were frozen at -80°C for at least 5 days for safety reasons and then kept at -20°C until used.

## Parasitological Methods

### Intestinal Scraping Technique

Small intestines were opened with gut scissors and visually inspected for the presence of *E. multilocularis* and other helminths. After removal of coarse intestinal contents, smear samples were taken from locations at 10 cm intervals by scraping the mucosa with glass slides and then pressing it on Petri dishes. The samples were examined using a stereomicroscope with magnifications of 4X to 40X. All procedures were performed with appropriate safety measures taken at site. The numbers and developmental stages of the recovered helminths were recorded. Their morphological features were drawn and studied using a light microscope equipped with a Camera Lucida apparatus.

### Sedimentation and Counting Technique

Small intestines were divided into four to six pieces of 20 cm length and opened longitudinally. Large helminths were removed directly, and then pieces of gut were immersed in normal saline and incubated at 37°C for 30 min. The mucosa was stripped between two pressed fingers. Twenty scrapes were also made with a spatula or microscope slides. Samples were then left to sediment for 20 min, and then washed two times or until the supernatant was clear. Amounts of 5–10 ml of precipitate were examined for parasites against a dark background in rectangular plastic dishes with a counting grid, using a stereomicroscope at 120X magnification. All collected parasites were stored in 70% alcohol (11).

### Stool concentration methods

Formol–ether technique and sucrose floatation method were performed according to Muller (12) and Gary (13), respectively.

### Statistical methods

SPSS 16.0.0 (SPSS Incorporation) was used for statistical analysis. Pearson's Chi Square tests were performed to determine the probable relation between different variables.

## Results

71.7% of examined animals were infected with at least with one helminth infection, but according to necropsy examination of 85 animal intestines (84 red foxes and one jackal), 96.4% animals harbored at least one helminth infection (Table 1).

**Table 1:** Prevalence of helminthic infections in different species of canids in Moghan plain, Northwestern Iran

	Status of Helminth Infection				Total (No.)
Canids	Negative		Positive		
	No.	%	No.	%	
Red fox	7	7.9	82	92.1	89
Dog	36	61	23	39	59
Jackal	0	100	1	100	1
Total	43	28.9	106	71.7	149

Based on necropsy methods, the most common helminth infection among red foxes of Moghan plain belonged to *Mesocestoides* spp. with the prevalence as high as 79.8% (Table 2).

**Table 2:** Prevalence of helminth infections among red foxes, dogs, and jackals in Moghan plain, Northwestern Iran

Helminths species	Red fox (n=89) No. (%)	Dog (n=59) No. (%)	Jackal (n=1) No. (%)	Total (n=149) No. (%)
*Mesocestoides* sp.	71 (79.8)	0 (0.0)	1 100	72 (48.3)
*Rictolaria* sp.	47 (52.8)	5 (8.5)	0 (0.0)	52 (34.9)
*Toxocara canis*	38 (42.7)	14 (23.7)	0 (0.0)	52 (34.9)
*Macranthorhynchus hirudinaceus*	39 (43.8)	2 (4.3)	0 (0.0)	41 (27.5)
*Toxascaris* sp.	30 (33.7)	9 (15.3)	0 (0.0)	39 (26.2)
*Joyeuxiella* sp.	29 (32.2)	0 (0.0)	0 (0.0)	29 (19.5)
Hookworms	18 (20.2)	1 (1.7)	1 (100)	20 (13.4)
*Taenia* spp.	10 (11.2)	2 (3.4)	0 (0.0)	12 (8.1)
*Alaria* sp.	2 (2.2)	0 (0.0)	0 (0.0)	2 (1.3)
*Tricuris* sp.	1 (1.1)	1 (1.7)	0 (0.0)	2 (1.3)
*Capillaria* sp.	0 (0.0)	1 (1.7)	0 (0.0)	1 (0.7)
Oxyuridae	1 (1.1)	0 (0.0)	0 (0.0)	1 (0.7)
*Dipylidium caninum*	1 (1.1)	0 (0.0)	0 (0.0)	1 (0.7)

**Fig. 2:**
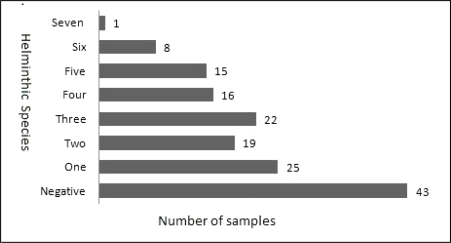
The status of multiple intestinal helminth infections among canids in Moghan plain area, Northwestern Iran (n=149)

54.3% of animals were infected with two or more helminths. The most intensive case, a male fox in Bileh Savar, was infected with 7 helminth species (Fig. 2).

There was a significant difference among the prevalence of helminth infections in three counties (χ^2^=21.973; df=2; *P*<0.001). All 36 samples (100%) from Bileh Savar County were infected with at least one helminth infection, whereas the rates in Pars Abad and Germi were 72.2% and 57.1%, respectively.

A significant difference was also observed among the prevalence of helminth infections regarding the sampling seasons (χ^2^=44.715; df=3; *P*<0.001). All the samples collected during the spring-harbored eggs, larvae or adult forms of at least one helminth species, while in summer, autumn and winter the rates were 94.3%, 44.8% and 52.9%, respectively.

The prevalence of *Toxocara. canis*, *Mesocestoides* sp., *Rictolaria* sp., *Macranthorhynchus hirudinaceus*, Hookworms and *Joyeuxiella* sp., infections differed significantly in different seasons. All the helminths showed the highest and lowest prevalence in summer and winter (Table 3). There was a significant relation between all aforementioned infections and the location of sampling so that the prevalence of *T. canis*, *Toxascaris* sp., *Mesocestoides* sp., *M. hirudinaceus* and *Joyeuxiella* sp., infections in Bileh Savar were higher than the two other counties (Table 4).

**Table 3:** Prevalence of helminth infections during different seasons in Moghan plain area, Northwestern Iran

Helminth species	Spring (%)	Summer (%)	Autumn (%)	Winter (%)	P value	χ^2^
*T. canis*	47.6	49.1	24.1	11.8	0.004	13.132
*Toxascaris* sp.	42.9	37.7	10.3	23.5	0.003	14.273
*Mesocestoides* sp.	85.7	84.9	15.5	0.0	0.000	81.054
*Rictolaria* sp.	66.7	45.3	17.2	0.0	0.000	20.770
*M. hirudinaceus*	33.3	45.3	17.2	0.0	0.000	18.268
Hookworms	38.1	15.1	6.9	0.0	0.001	15.889
*Joyeuxiella* sp.	19.0	47.2	0.0	0.0	0.000	44.083

**Table 4:** Prevalence of intestinal helminth infections in three counties; Pars Abad, BileSavar and Germi

Helminth species	Pars Abad (%)	Bileh Savar (%)	Germi (%)	P value	χ^2^
*T. canis*	33.3	69.4	19.5	0.000	27.005
*Toxascaris* sp.	36.1	58.3	6.5	0.000	36.541
*Mesocestoides* sp.	41.7	94.4	29.9	0.000	48.804
*Rictolaria* sp.	30.6	55.6	27.3	0.011	9.031
*M. hirudinaceus*	22.2	50.0	19.5	0.002	12.123
Hookworms	5.6	25.0	11.7	0.044	6.269
*Joyeuxiella* sp.	16.7	58.3	2.6	0.000	48.853

### Results of necropsy methods

The prevalence of identified worms were *Mesocestoides* sp. 84.7%, *Rictolaria* spp. 55.3%, *M. hirudinaceus* 45.9%, *T. canis* 43.5%, *Toxascaris* spp. 35.3%, *Joyeuxiella* sp. 34.1%; hookworms (either *Ancylostoma caninum* and/or *Uncinaria stenocephala)* 22.4%, *Taenia* spp. 11.8% (including 6 cases of *Taenia polyacantha*, 3 *Taenia endothoracica* and 1 *Taenia hydatigena*), *Alaria* spp. 2.4% and *D. caninum* 1.2%. The intestine of the jackal harbored adult worms of *Mesocestoides* sp. and Hookworms as well. Based on the necropsy methods, 96.5% canids harbored the adult worms of at least one parasitic helminth species. Total helminth infection rates in three counties showed no significant difference but the prevalence in Bileh Savar was the highest (100%).

*T. canis* prevalence in Germi was significantly lower than Bileh Savar and Pars Abad and the in Bileh Savar was higher than Pars Abad (χ^2^=26.230; df=2; *P*<0.000). The same status was observed with *Toxascaris* Sp. (χ^2^=25.257; df=2; *P*<0.000) and *Joyeuxiella* sp. (χ^2^=18.893; df=2; *P*<0.000) infections. Furthermore, the prevalence of *Mesocestoides* sp. and *Ricltolaria* sp. differed in three counties, but the differences were only statistically significant in confidence intervals of 90% (*P*=0.090). Multiple infections in three counties differed significantly so that the rate in Bileh Savar was higher than Pars Abad and Germi (*P*=0.02).

Among all the helminthes, only prevalence of *Rictolaria* sp. (χ^2^=9.348; df=1; *P* value= 0.002) and *M. hirudinaceus* sp. (χ^2^=4.927; df=1; P<0.026) showed a significant difference in genders, as the male animals had higher infection rates.

### Results of stool concentration methods

30.9% of samples were infected with eggs of at least one helminth species, ten animals harbored eggs of 2 helminth species, six had eggs of 3 species and one had eggs of 5 species. The prevalence of helminth infection among dogs and foxes was 37.3% and 26.6%, respectively but the difference was not statistically significant. Sampling season had no influence on the results. The prevalence of helminths among red foxes of Bileh Savar county was significantly higher than those of Pars Abad and Bileh Savar counties (χ^2^=7.407; df=2; *P*<0.05). Details are reflected in Table 5.

**Table 5:** Prevalence of helminth infections among canids in Moghan Plain based on detection of eggs in fecal samples

Helminth species	Red fox (n=89) No. (%)	Dog (n=59) No. (%)	Total* (n=149) No. (%)
*Toxascaris* sp.	10 (11.2)	11 (18.6)	21 (14.1)
*T. canis*	6 (6.7)	10 (16.9)	16 (10.7)
*Rictolaria* sp.	7 (7.9)	5 (8.5)	12 (8.1)
Acanthocephala	11 (4.12)	2 (3.4)	13 (8.7)
*Tricuris* sp.	5 (5.6)	1 (1.7)	6 (4.0)
*Taenia* spp.	1 (1.1)	2 (3.4)	3 (2.0)
*Mesocestoides* sp.	3 (3.4)	0 (0.0)	3 (2.0)
Hookworms	0 (0.0)	1 (1.7)	1 (0.7)
*Capillaria* sp.	0 (0.0)	1 (1.7)	1 (0.7)
*Fasciola* sp.	0 (0.0)	1 (1.7)	1 (0.7)

* There was no helminth egg in the Jackal fecal sample

## Discussion

The result of this survey showed that the most common helminth infection belonged to *Mesocestoides* sp. This helminth has already been reported in jackals, red foxes, wolves, stray, and sheep dogs from different parts of Iran (2, 4, 5, 8, 14). The presence of this helminth has also been documented in other countries including Belarus, Portugal, and Italy with prevalence rates of 13.8%, 30.2%, and 26.6% respectively (15–17). However, in a study on 588 red foxes in Great Britain, no animal was shown to harbor this parasite (11). The prevalence of this helminth in our study was 79.6%, which was higher than all previous reports except one from western part of Iran (2). Intensity of the infection, determined by a subjective method, was high in a considerable number of animals so that 23.6% and 19.1% of samples were 3+ and 4+, respectively. The infection rates during the summer and in Bile Savar County were significantly higher than other seasons and counties. The higher prevalence of *Mesocestoides* sp. infection in this study, in comparison to the previous ones, may be due to food resources that are available for red foxes. It seems that coprophagic mites, as the first intermediate host, and amphibians, reptiles, small mammals and birds, especially home-breaded chickens, as the second intermediate hosts for parasite, are a major part of red foxes' diet.

In necropsy of red foxes, 44 out of 84 (52.4%) were infected with animal ascarids, the rate among domestic dogs was lower (32.2%) which might be due to type of samples (stool) and examination method. These worms are considered as the most important causative agents of visceral larva migrant (VLM) disease in humans (12). The prevalence of these worms in our study was higher than most of previous ones (2, 5)614, even in domestic dogs in which stool samples were examined rather than intestines.

The prevalence of hookworms' infection including *A. cannium* and *U. stenocephala* among red foxes was 21.4% and the only jackal studied showed a heavy infection. In a previous study on carnivores in the same region, the prevalence rate of *A. cannium* and *U. stenocephala* infections among red foxes were 8.6% and 21.4%, respectivlely (19). In addition, Dalimi et al. showed that prevalence of *A. cannium* and *U. stenocephala*, among red foxes in western parts of Iran were 4.5% and 13.6%, respectively (2).

*Joyeuxiella* sp. infection among red foxes has previously been reported from the same area by Mobedi et al. (3) and Zariffard (5) with prevalence of 46% and 15.7%, respectively; but the prevalence of *D. caninum* in the two study were 0.5% and 1.4%, respectively (5, 20). Moreover, 7.7% of red foxes and 30% of jackals from western provinces of Iran were infected with *Joyeuxiella pasqualei* (2).

As seen in the present study and most of the previous reports, there is a big difference in the profile of *Joyeuxiella* sp. and *D. caninum* infections in red foxes and dogs, i.e. the prevalence of *Joyeuxiella* sp. among red foxes was higher than that of dogs while *D. caninum* among dogs was of higher prevalence. The difference might be due to different life cycles of the parasites. It has been shown that *Joyeuxiella* sp. utilizes reptiles, especially lizards, as the second intermediate hosts, which are probably main components of red foxes diet. There is no report of *Joyeuxiella* sp. infection in red foxes of European countries including Belarus (17), Great Britain (11), Italy (15) and Portugal (16), which is probably resulted from different climatic conditions and animal fauna in these countries.

In this study, *Acanthocephala* infection showed a high prevalence among red foxes as 46.6%. The acanthocephalan species was diagnosed as *M. hirudinaceus* based on morphologic features. In the previous reports, the prevalence of *M. hirudinaceus* was 10-23% (5, 2). In Belarus 3.2% and in Portugal 1.6% of red foxes were infected with Acanthocephalan species (16, 17). However, in two separated studies in Great Britain and Italy, no acanthocephalan helminth was found (11, 15).Presence of insects' residues, especially Scarabid beetles, in the intestines would be an explanation to the high prevalence of *Acanthocephala* sp. infection among red foxes in Moghan plain.

*Rictularia* sp., (now *Pterygodermatites*), the sole genus in the superfamily of Rictularioidea (Order: Spirurida), were found in 56% of red foxes. In the life cycle of this intestinal worm arthropods, especially cockroaches, and Entomophagous vertebrates such as lizards play the role of intermediate and paratenic hosts, respectively and carnivores as final hosts are infected via ingestion of these animals. The worm is distributed in Africa, east and south Europe, Middle East and India (21). In the previous studies in Iran, 45% of red foxes in Ardebil Province (5), 1% of cats in Khuzestan Province (6) and 54.5% of red foxes, 12.5% of dogs and 1% of jackals in western provinces of Iran were infected with *Rictularia affinis* (2). Moreover, 24.2% of red foxes from a district in Italy were infected with *Pterygodermatites affinis* (15) and 3.23% and 6.45% of red foxes from Portugal harbored *P. affinis* and *R. proni*, respectively (16). However, in two studies on helminth fauna of red foxes in Belarus and Britain this helminth was not observed (11, 17). As discussed earlier, the high prevalence of this worm may be a reflection of food sources, i.e. arthropods and entomophagous vertebrates that are available for red foxes in the area.

*Alaria* sp. was the only parasitic trematode species that we could recover. *Alaria* sp. infections were previously reported from jackals and dog in Caspian Sea littoral (14), jackals in western provinces (2) and red foxes in this area (5). The prevalence of *A. alata* in Italy and Portugal showed to be 24.2% and 27.4%, respectively (15, 16). The lower prevalence of *Alaria* spp. in Iran, compared to European countries may probably be due to less favored environmental conditions for growth and survival of snails that serve as first intermediate hosts.

The lower prevalence of *Mesocestoides* sp., *Rictolaria* spp., *T. canis*, *Joyeuxiella* sp., and *Toxascaris* spp. infections in Germi in comparison to Bileh Savar and Pars Abad is probably due to high altitude and cold climate condition. The other two counties are warmer and are commonly utilized as winter resorts by completely nomadic populations of Ardebil Province. The lower total prevalence of helminth infections in autumn and winter compared to spring and summer could be resulted from decreased populations of intermediate or paratenic hosts especially arthropods and lagomorphs in cold seasons on which the canids pray.

This study showed that various helminthes, especially soil transmitted species and causative agents of cutaneous larva migrans (CLM) and VLM syndromes in humans are of high prevalence among canids of the Moghan plain. High humidity of the area resulting from relatively high annual precipitation (approximately 300 mm) and vicinity to Caspian Sea may provide a suitable environmental condition for growth and survival of eggs and larvae of the soil-transmitted helminths. Considering the high prevalence and intensity of the helminths infection among canids as well as numerous world reports on human cases, an investigation on medical and veterinary importance of these zoonotic infections in the area seems to be necessary.
